# Enhancing trust in news media: A multimodality approach to detecting fake news with social constructs

**DOI:** 10.1371/journal.pone.0339793

**Published:** 2025-12-31

**Authors:** Ishfaq Ali, Muhammad Asif, Osama Sohaib, Ahmet Savaşan, Hanan Aljuaid

**Affiliations:** 1 Department of Computer Science, National Textile University, Faisalabad, Punjab, Pakistan; 2 Department of Statistics and Business Analytics, College of Business and Economics, United Arab Emirates University, Al Ain, United Arab Emirates; 3 School of Computer Science, University of Technology Sydney, Australia; 4 Department of Sports Science, Near East University, Nicosia, Mersin, Turkey; 5 Computer Sciences Department, College of Computer and Information Sciences, Princess Nourah bint Abdulrahman University (PNU), Riyadh, Saudi Arabia; Tarbiat Modares University Faculty of Medical Sciences, IRAN, ISLAMIC REPUBLIC OF

## Abstract

The widespread dissemination of misinformation and fake news on social media platforms poses a significant challenge to the integrity of public discourse, democratic stability, and societal trust. Although misinformation detection has been widely studied, one critical but underexplored dimension is the misquotation of legitimate news articles on social media. Misquotations—deliberate or accidental distortions of original news content—can mislead audiences and diminish trust in credible news sources, thereby amplifying the reach of misinformation. Addressing this issue requires a robust dataset that captures the original news content and its associated social media representations. To this end, we construct a comprehensive dataset by performing multi-source triangulation across four established datasets: FAKENEWSNET, NELA-GT, TruthSeekers, and Twitter15/16. This process yields approximately 158,400 aligned pairs of news stories and related social media posts. Building on this dataset, we propose a multimodal binary classification framework designed to detect misquotations by jointly modeling textual, visual, and contextual features. The model integrates engineered features representing social context and event semantics within a shared latent space, enabling a nuanced understanding of content distortion. Furthermore, we analyze the relative contribution of each modality—textual, visual, and contextual—using ablation studies and performance metrics to assess their impact on detection accuracy. This study introduces a novel approach to misquotation detection, contributing to the combat of misinformation through multimodal analysis.

## Introduction

Misquotation of news media online, where content is doctored or falsely attributed, erodes trust in mainstream outlets. Social media algorithms amplify these distortions, spreading them through shares and memes that mimic credible sources, such as leading news media channels [1,2]. Experimental data show that exposure to misquoted headlines fosters a distrust mindset, reducing confidence in all news [1,3–5]. Correlational studies link such exposure to lower media trust, fueling polarization [6]. Yet, research lacks focus on platform-specific mechanisms, such as X’s quote-tweeting. Targeted studies are needed to design effective interventions, such as enhanced fact-checking [7,8] The misquotation of news media online significantly undermines trust in mainstream sources, with profound implications across multiple domains. For media businesses, eroded trust reduces audience engagement, leading to decreased subscriptions and advertising revenue as resources shift to unverified platforms [1,9]. Business organizations face reputational damage and decision-making errors due to distorted information environments. In state affairs, misquotations weaken public confidence in governance, destabilizing democratic processes [8,10]. Public health is compromised by misinformation, notably vaccine hesitancy, and increasing morbidity rates [11]. Politically, misquotations exacerbate polarization and erode electoral integrity, fostering voter skepticism [12,13].

Traditionally, misinformation detection strategies have focused on either content verification or social context analysis [14]. Content verification methods typically employ natural language processing (NLP) techniques to identify inconsistencies within textual content or assess the credibility of sources. Models such as BERT (Bidirectional Encoder Representations from Transformers) have been pivotal in this domain [15], enabling the detection of manipulation patterns in text. However, these approaches often overlook the significance of the social context in which misinformation proliferates.

Conversely, social context analysis emphasizes the behavior of users and the dynamics of information dissemination within social networks. Techniques that examine engagement patterns, such as the presence of bots or echo chambers, provide valuable insights into the mechanisms driving the spread of misinformation. However, relying solely on social context can lead to incomplete assessments, as it does not account for the inherent characteristics of the misinformation itself [14–16].

Given the limitations of existing methodologies, there is a growing recognition of the need for a multimodal approach that integrates both content verification and social context modeling [14]. By combining these dimensions, researchers can develop a more comprehensive understanding of misinformation dynamics. This study proposes a novel framework that integrates textual and visual content analysis with social engagement metrics, facilitating a more nuanced detection of misinformation [17,18].

A critical aspect of this research is the development of a triangulated dataset comprising 158,400 instances that link news articles to their social media derivatives. This dataset is enriched with veracity labels and multimodal features, providing a rich foundation for training and evaluating our proposed framework. The creation of this dataset not only facilitates rigorous testing of the framework but also contributes to the broader research community by providing a valuable resource for future studies. A metric for context sensitivity is created based on the context-sensitivity, and the event score is calculated from the cluster density. These engineered features facilitate the deployment of multimodal machine learning, enabling the assessment of each modality.

This study makes several key contributions to the field of misinformation research. First, it advances the understanding of how content and context interact in the propagation of misinformation. By unifying these elements, we provide a comprehensive framework that enhances detection capabilities. Second, the framework’s explainability features enable the quantification of modality contributions, which can be invaluable for researchers and practitioners seeking to understand the underlying factors driving the dissemination of misinformation. Lastly, the real-world viability of the framework, as demonstrated by its rapid processing times and high precision in detecting coordinated campaigns, highlights its potential for practical applications in combating misinformation.

As misinformation continues to pose significant challenges in various domains, the need for effective detection systems is more pressing than ever. This study introduces a novel multimodal framework that integrates content verification and social context modeling, providing a comprehensive approach to misinformation detection. By leveraging advanced methodologies and a rich dataset, this research contributes to the ongoing efforts to understand and mitigate the impact of misinformation in our increasingly digital world. Future work will focus on refining the framework, exploring additional modalities, and enhancing its applicability in real-world scenarios, ultimately striving to foster a more informed society.

## Literature review

The concept of multimodality, to many authors, is different; for some authors, it is a fusion of multimedia (audio, video, and text), while for others, it is the fusion of context and content [17,18]. Let us examine the definition of multimodality in the context of fake news detection as presented by different authors. The first practical approach with multimodality and deep learning fusion was [19], which used RNN for data fusion. The EANN Model [3] It is a KDD-2018 award-winning research project that defined multimodality as a combination of context and content. The contents include textual and visual features. Their prestigious research work was one of a kind in terms of catering to the context-of-event and implementations of generative adversarial networks for FND tasks. Recently, a master’s thesis presented the broadest range of multimodality [20]. A simple bird’s-eye view suggests that most authors consider text and visual features to be the most significant for detecting fake news. The other reason for Wiebo and the Twitter dataset was that it synthesized both text and visual information [13,21]. If we critically analyze the available datasets, most of them are based on text, visual, and meta information that seem to be inherent features of the datasets. Considering that “Event” in fake news detection is rare, only two of the entire start-sets practically used the Event in their multi-modality-based approach. However, the essence of fake news propagation is significantly related to events; two notable examples are the 2016 US Election and the COVID-19 pandemic [22,23]Origin generated a tremendous amount of fake news over social platforms.

The argument is that each piece of news is based upon an event, and so is fake news. Therefore, fake news detection techniques must consider the Event as a potential candidate for a multi-modeled solution [24]. Then, why most researchers prioritize text, visual, and metadata features for their project can be explained by the fact that available datasets are not synthesized in such a fashion. Therefore, most authors preferred visual and textual data with fusion metadata or context information. Event consideration by EANN [3] Simulates a method for synthesizing event-centered data using Generative Adversarial Networks (GANs), the first application of generative adversarial networks in the FND domain [20]. Moreover, their work utilized pre-trained models, specifically VGG-19, which is another notable innovation in the field of FND research. Another contemporary data mining approach proposed memorizing events. The authors presented a novel Multimodal Knowledge-aware Event Memory Network (MKEMN) [25,26] To detect rumors on social media by integrating the Multimodal Knowledge-aware Network (MKN) with the Event Memory Network. It was done to make the MKEMN as effective as possible (EMN). The authors present a reliable method for detecting events that have been the subject of rumors on Twitter. Their findings justify a new area of study: event-centered multi-model detection of fake news. Text summarization and visual summarization may be used to identify events from available datasets, which may need multi-domain expertise. To label textual and visual elements into a specific event, however, a deep learning method employed by EANN [3] Based on generative adversarial networks, it provides an alternative and superior solution to the summarizing strategy. A unique approach synthesizes event-centric data from social media postings, primarily utilizing text data. It also suggests using a visual summarizing method to do this. Their technique, called Event-Bagging, offers a framework for visual information based on events.

Fake News Detection (FND) approaches can be categorized into three directions: user-posted data, government/ law enforcement agencies, and social media platform administrations [27]. The user-posted data provides statistical grounds for FND; hence, it widely utilizes machine learning and deep learning [28,29]. The user-posted data approaches have two further divisions, i.e., pattern-driven practices and content-centered methods. The scope of this research is delimited to a user-centered approach. The content-centered strategies are based on text, visual, context, Event, and multimedia approaches.

The multimodal features-based FND approach’s argument is more effective than the text-only approach because each news story posted on social media platforms is multimedia-based [30,31]. Some authors trained their models independently for various features, whereas others used a fusion of multimedia. The scope of research is delimited to approaches with a fused multimodal approach. The following paragraphs provide a summary of how multi-modeled techniques evolved over the years. The very first significant approach for the fused multi-modeled process. Their system catered to both textual and contextual information, jointly represented across modalities. Their deep learning approach is also augmented with visual features. This study features the most diverse dataset, utilizing two datasets: the Twitter and Weibo datasets.

The authors participated in the KDD-2018 challenge, one of the prestigious challenges in applied data science. This work can serve as a first approach to detecting fake news in the context of events [32–34]. The work addresses one of the unique challenges in fake news detection on social media: identifying fake news related to newly emerging events. Previously, approaches tended to learn event-specific features that cannot be transferred to unseen events. Datasets prepared for fake news deception are used to train models in a specific context. Models perform poorly for newly emerged incidents. It was also the first approach to implement Generative Adversarial Networks (GAN), debunking misinformation over social media [3,35]. The following paragraph summarizes their approach. EANN consisted of one generator, two discriminators, and an integrator. The multi-modeled generator offers a mechanism for fusing textual information using [107] and visual information utilizing the VGG-19 technique. To determine whether the posts are authentic, a fully linked layer equipped with a softmax and an event discriminator that separates visual from textual information is implemented. The output of the multimodal feature extractor is used as the input for the fake news detector, which is constructed on top of the multimodal feature extractor. The purpose of the fake news detector is to ascertain whether or not it contains false information concerning each given post. Their efforts could be viewed as a singular addition to the event-centric strategy. Their method, nonetheless, falls short in terms of event-generations verification.

Mr Gao [36–38] and his colleagues conducted a research series utilizing different multimodal features. Their work simulates other statistical research grounds for FND approaches. Their work includes machine learning and deep learning approaches. One of his students further carried out their work. This series of work provides machine learning and deep learning approaches with multimodal implementation. Another contribution is the utility of pre-trained models. Another series of deep learning models for fake news detection, specifically SPOTFAKE and SPOTFAKE + , was introduced in 2019−20 [39,40]. These models utilized a pre-trained model, namely BERT [41], for text datasets and VGG-19 [42] for image datasets. A key contribution of their work is countering the ENN technique, which treats the Event as a sub-task, discriminating events from data rather than a full-fledged feature for FND tasks. However, their approach strictly defined multimodality in Text and Image only. The criticism of their work is that their approach is essentially based on the Twitter dataset, which is synthesized over text and images only (videos and audio are excluded).

The SPOTFAKE+ [39] modeled over the catering critics of the least featured dataset for multimodality to identify misleading information using the SPOTFAKE dataset with additional information [40]. Meanwhile, another approach used a multimodal auto-encoder technique for fake news detection (opened another deep learning direction for fake news detection). The same set of datasets was used by [41,42], and their model outperformed by ~6% in accuracy and ~5% F1 score. The Multimodal Variational Autoencoder, often MVAE [43] It is a full-stack network explicitly developed to detect fake news. This network incorporates both a bimodal Variational auto-encoder and a binary classifier within its structure. The model comprises three fundamental components: an encoder, a decoder, and a module for identifying false information. The Variational autoencoder can generate probabilistic latent variable models by maximizing a bound on the marginal likelihood of the observed data [44]. These models can be used to make predictions. It enables the model to have a higher degree of precision. To determine whether posts are genuine, the fake news detector consults the multimodal representations generated by the bimodal Variational Autoencoder [25] r. It helps the detector avoid making mistakes. Another approach, which incorporates an additional textual feature — user sentiment —for consideration in fake news detection. They utilize users’ comments on a particular social media post or blog [28,29]Their approach’s uniqueness includes features that collectively cluster events, i.e., Topic, Keywords, Authors, and other meta information.

The very significant approach [45] That caters to visual information, and the annotation of video data can be considered a benchmark approach for FND tasks. Their approach is more straightforward, considering the similarity between video and text annotation. Their approach is based on mainstream news media stories in both textual and visual formats, i.e., longer manuscripts and longer videos, unlike social media-posted data. So, we may consider this study to induce video as another potential feature. Exploring deep neural networks [46] The rumor introduces another feature, i.e., context. This study introduced a feature of context in which news stories are covered, as most journalists write stories or share vlogs within a specific context. Therefore, context must be considered for FND. A political context [47] is one of the most researched contexts in FND techniques [48,49]. Based on studies, multimodality is defined as the integration of textual, visual, and acoustic information (audio features). The detection of fake news on social media, as inferred from current advancements, principally stems from the complex interaction of multimedia data, social context, and the relevant social and political events. This comprehensive method acknowledges that language analysis alone is often insufficient, requiring a thorough understanding of visual material, user interactions, and the broader societal context to identify deception effectively.

## Proposed methodology

**Fig 1:**
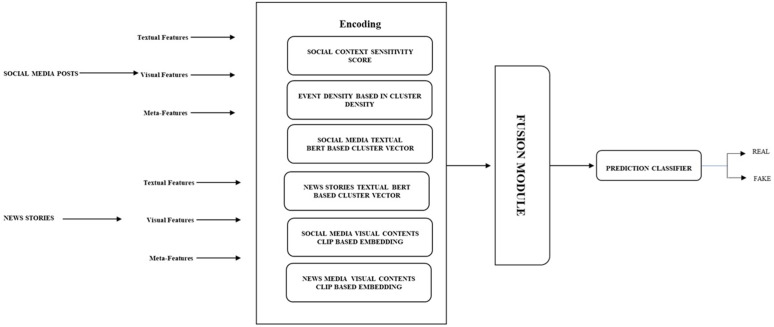
Proposed multi-modal process.

The entire research process encompasses a literature search and state-of-the-art research, formulating research objectives, and selecting empirical evidence. The next part involves the collection of datasets, including the identification of relevant datasets and the merging or normalization of these datasets, as well as the development of a representation scheme for diverse data sources. According to the experimental design, both joint and disjoint representation schemes for modalities are proposed. The next step is designing encoders for each modality, followed by training. The results of each fusion scheme are recorded, and a comparison is performed. Lastly, ablation studies are proposed to identify the contributions of each modality. The entire process is described in the following subsections. The proposed method is given in Fig 1.

### Multi-sourced triangulation process

#### Multi-sourced triangulation of data.

The triangulation process is initially performed on the selected datasets, as given in Table 1, and approximately 158,400 instances are filtered.

**Table 1:** Data sources.

Dataset	Content Type	Size	Key Attributes	Source
NELA-GT-2023	News articles	72,000	Publisher metadata, credibility labels	Harvard Dataverse
FakeNewsNet	Social posts + images	86,000	Image-text pairs, engagement metrics	ASU Repository
Twitter/X/X 15–16	Social media threads	210,000	Retweet graphs, timestamps, and bot scores	Twitter Developer Portal
TruthSeekers	Fact-checked claims	12,000	Debunking reports, manipulation tags	Google Fact-Check Tools

**Fig 2:**
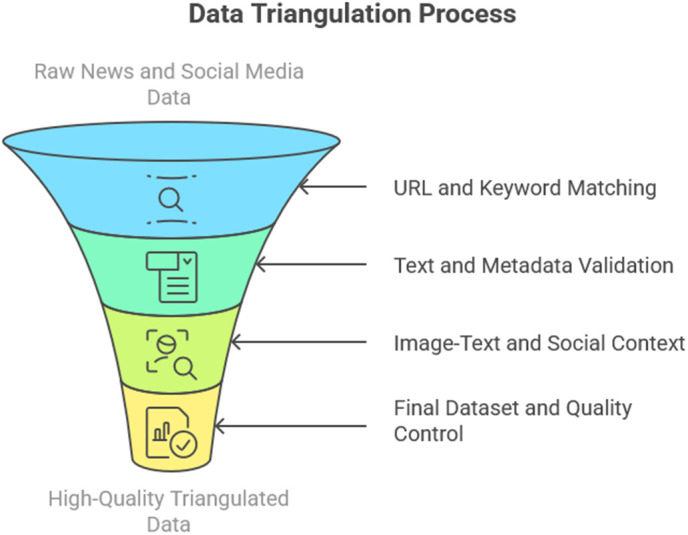
The triangulation process.

The Triangulation Process delineates a systematic, multi-faceted approach to authenticate and synchronize data from news articles and social media posts, thereby ensuring reliability and reducing bias. The complete procedure is illustrated in Fig 2

The Triangulation Process is a robust, multi-step approach designed to thoroughly align and validate data from social media posts and news articles, thereby significantly increasing reliability and reducing inherent biases. The first step is URL and keyword matching. The algorithm first ensures direct correlation by giving priority to exact domain matches for URLs, such as bbc.com/news. To account for minor deviations while preserving accuracy, shortened links are allowed to have a Levenshtein distance of 5 or less. When URL pairs are out of alignment, the focus switches to keywords. In this case, latent topic clusters are identified by the potent language model BERT. Even if sources have different URLs, only couples with a high cosine similarity—more precisely, a value of≥0.85—are retained, ensuring that their main topic remains consistent.

The next critical phase is text and metadata validation. For textual content, BERTScore is employed to detect subtle yet significant distortions, such as sentiment flips (e.g., a positive statement becoming negative) or outright entity swaps (e.g., “Biden” incorrectly replaced with “Trump”). Simultaneously, metadata undergoes strict scrutiny. Timestamps are meticulously checked to ensure that news articles consistently precede their corresponding social media posts, establishing temporal precedence. Furthermore, MD5 hashes are utilized to identify any unauthorized image edits, safeguarding visual integrity.

Following this, Image-Text & Social Context are thoroughly analyzed. CLIP, an advanced neural network, measures the semantic alignment between images and accompanying text; pairs with a similarity score below 0.7 are discarded to prevent misleading visual-textual combinations. The social context is then enriched by assessing bot likelihood using the Botometer API, analyzing virality through retweet graphs, and identifying sudden temporal bursts in activity via Poisson fitting, which provides deeper insights into the dissemination and potential manipulation of the information.

**Fig 3:**
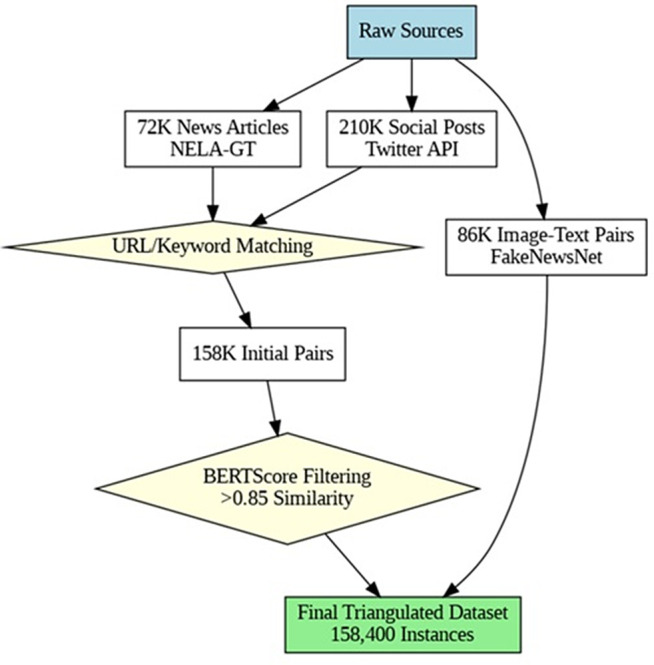
Triangulation workflow.

Finally, the Final Dataset & Quality Control stage aggregates all triangulated data. This includes a crucial manual annotation step, where five annotators label 5,000 samples, achieving a substantial inter-annotator agreement (κ = 0.81), with any disputes resolved by consensus. The dataset is then strategically split into 70% for training, 15% for validation, and 15% for testing, with stratification by publishers to ensure representativeness. A proactive bias mitigation effort balances political leanings, aiming for approximately 42% left, 38% right, and 20% neutral content, providing the dataset’s impartiality. This comprehensive integration of NLP, network analysis, and human oversight ensures high-quality, cross-verified data, which is essential for robust analysis. Fig 3 explains the entire process and workflow for the triangulation.

This pseudocode outlines a comprehensive pipeline for constructing a high-fidelity, multimodal fake news detection dataset by triangulating information from verified news articles and social media posts. The process includes URL and topic matching, content validation, image and context verification, and socio-political balancing. Each step is designed to enhance dataset quality by ensuring semantic alignment, detecting manipulation, and incorporating human annotation and bias control.

## Proposed multimodal classifier

### Engineered features

#### Social context sensitivity.

Characteristics of the social context significantly enhance the resilience and reliability of classification algorithms for detecting fake news. Social interaction dynamics can provide essential evidence for assessing the veracity of online material, despite textual and visual modalities being the primary indicators of credibility. The Context Sensitivity Score (CS) is a socially-informed statistic that quantifies the social propagation characteristics of content, particularly concerning ideological uniformity and automated dissemination.

The basis of social context sensitivity is the understanding that misinformation often disseminates differently than genuine content. Echo chambers reinforce belief systems through selective exposure and suppression of dissenting perspectives, while bots are frequently utilized to disseminate disinformation campaigns. Consequently, significantly separated groups or content predominantly spread by automated agents often exhibit distinct propagation patterns indicative of manipulation.

To quantify these effects, we define the Context Sensitivity Score (CS) as given in equation 1:


CS=0.6·σ(bot_score)+0.4·echo_chamber (1)

Where:

- $\sigma (x)=\frac{\text{1}}{\text{1}+e^{-x}}$ The sigmoid function is used to normalize the bot’s likelihood score into the [0, 1] range.
- bot_score∈[0,1] represents the likelihood that the user posting or amplifying the content is a bot, typically derived from account metadata (e.g., activity frequency, follower/following ratios, linguistic patterns).

echo_chamber∈[0,1] reflects the ideological or community isolation of the content’s dissemination path, computed via graph-theoretic metrics such as modularity or inter-cluster interaction ratios in user engagement networks.

The weighting factors of 0.6 (for bot likelihood) and 0.4 (for echo-chamber isolation) were determined through empirical experimentation and hyperparameter tuning on a dedicated validation split (15% of the training data, 23,760 instances). Ablation studies showed that this ratio consistently yielded the best F1-score compared to alternatives such as 0.5/0.5 or 0.7/0.3.

The weighted formulation, with coefficients of 0.6 and 0.4, respectively, prioritizes bot activity based on empirical findings that automated accounts disproportionately contribute to the spread of misinformation. The echo chamber component, while secondary, captures the socio-political silos within which fake news often thrives.

### Social events

An event is characterized as a cohesive collection of user-generated posts that demonstrate temporal, semantic, and possibly physical closeness in the context of social media-driven fake news detection. These postings are usually focused on a particular real-world incident, topic, or story. Clustering algorithms are used to identify events by grouping posts based on common characteristics, such as the time of publication, textual similarity (e.g., shared keywords, subjects, or hashtags), and, if available, geographic metadata.

Formally, an event E can be represented as given in equation 2:


$$E=\{p_{\text{1}},p_{\text{2}},...,p_{n}\}\;\text{where}\;\forall p_{i}\in E,\;\text{Sim}(p_{i},p_{j})\geq \theta \;\text{and}\;|t_{i}-t_{j}|\leq \Delta t$$ (2)

Where:

- $p_{i}$ and $p_{j}$ are individual posts,
- Sim(·) is a similarity function (e.g., cosine similarity, BERT embedding similarity)?
- θ is the semantic similarity threshold,
- Δt is the maximum allowable time difference within the event cluster. To capture this dimension, we define the EventScore, a scalar feature designed to quantify the intensity and spatial coherence of a social media event over time.

The following equation gives the EventScore given in equation 3:


EventScore=post_rate·ln(cluster_density+1) (3)

Where:

- $\text{post}\_\text{rate}=\frac{\text{total}\;\text{posts}\;\text{about}\;\text{event}}{\text{time}\;\text{window}\;(\text{e}.\text{g}.,\;\text{per}\;\text{hour}\;\text{or}\;\text{day})}$ captures the velocity of information propagation, which is often higher for misinformation due to coordinated bot activity or virality.
- $\text{cluster}\_\text{density}=\frac{\text{number}\;\text{of}\;\text{posts}}{{\text{spatial}\;\text{area}\;(\text{e}.\text{g}.,\;\text{in}\;\text{km}}^{\text{2}})}$ represents the geographical concentration of posts related to the event, identifying if the event is regionally isolated or globally dispersed.
- ln(·) is the natural logarithm, and the additive term (+1) ensures numerical stability, preventing undefined values when cluster_density=0.

Missing values for credibility_score, reach_index, and temporal_proximity (e.g., when metadata is unavailable) are imputed with median values from the training set or set to zero where appropriate; full details are provided in Section “Dataset splitting and preprocessing”.

### Normalized textual feature

Textual features are a fundamental modality in the identification of false news because they capture pragmatic, syntactic, and semantic information that is essential for spotting modified or misleading narratives. The suggested paradigm takes into account two primary textual information sources: (i) news media article text and (ii) social media post text. Pre-trained language models are used to process each of these inputs separately, producing high-dimensional embedding vectors that allow for comparison and fusion with other modalities later on.

(i)
**Social Media Post Text Vector**

Social media posts are typically short, informal, and highly context-dependent, often characterized by colloquial language, emotive expressions, and the use of hashtags. For each social media post $s_{i}$, we obtain a dense vector representation using a pre-trained transformer-based encoder such as BERT, given in equation 4:


$${𝐯}_{\text{social},i}={\text{BERT}}_{\text{CLS}}(s_{i})\in R^{d}$$ (4)

Where:

- $s_{i}$ is the $i^{\text{th}}\;$ social media post.
- ${\text{BERT}}_{\text{CLS}}(\cdot )$ Extracts the contextual embedding from the [CLS] token, representing the entire post.
- d is the embedding dimensionality (typically 768 for base models).

In certain instances, sentence-level embeddings (e.g., through Sentence-BERT or mean pooling of token vectors) are employed to improve semantic representation, particularly for longer postings or those containing sub-sentences. These embeddings encapsulate lexical characteristics, sentiment indicators, and stylistic tendencies commonly associated with disinformation.

(ii)
**News Media Article Text Vector**

News articles represent structured, formal texts designed to convey factual information. Each article $a_{j}$ is tokenized into a sequence of sentences $a_{j}=\{s_{\text{1}}^{j},s_{\text{2}}^{j},\ldots ,s_{n}^{j}\}$, and each sentence is encoded individually using the same BERT-based encoder. The final article-level vector is computed via mean pooling over sentence embeddings given in equation 5:


$${𝐯}_{\text{news},j}=\frac{\text{1}}{n}\sum_{k=\text{1}}^{n}{\text{BERT}}_{\text{CLS}}(s_{k}^{j})\in R^{d}$$ (5)

Where:

- $a_{j}$ is the $j^{\text{th}}$ news article.
- $s_{k}^{j}$ is the $k^{\text{th}}$ sentence in the article $a_{j}$.
- n is the number of sentences in the article.

### Visual feature representations

These embeddings encapsulate visual semantics, object-level context, and compositional attributes that empower the model to discern indications of visual deceit or misinformation (e.g., inappropriate stock photo usage, repurposed content, emotionally charged images).

We distinguish between two sources of visual input:

(i) Social Media Visual Vector

Social media posts often feature images, memes, or manipulated visuals that boost emotional impact or credibility. Each image attached to a post is processed using a pre-trained CLIP image encoder given in equation 6:


$${𝐯}_{\text{image},s}={\text{CLIP}}_{\text{img}}(I_{s})\in R^{d_{\text{2}}}$$ (6)

Where:

- ${\text{CLIP}}_{\text{img}}$denotes the image encoder component of CLIP.
- $d_{\text{2}}$ is the visual embedding dimension (e.g., 512 or 768, depending on model size).

These embeddings encapsulate visual semantics, object-level context, and compositional attributes that empower the model to discern indications of visual deceit or misinformation (e.g., inappropriate stock photo usage, repurposed content, emotionally charged images).

(ii) News Media Visual Vector

Similarly, if a news article includes an image $I_{n}$, It is encoded via the same CLIP encoder given in equation 7:


$${𝐯}_{\text{image},n}={\text{CLIP}}_{\text{img}}(I_{n})\in R^{d_{\text{2}}}$$ (7)

This representation captures the referential visual semantics typically aligned with the article’s narrative. These vectors serve as baselines for evaluating whether social media visuals are manipulated, unrelated, or distorted derivatives of the source.

(iii) Visual-Textual Semantic Alignment

To detect image-text inconsistencies, we compute the CLIP-based similarity between the image and its corresponding text caption or body (social or news) as show in equation 8:


$$\text{CLIPSim}(I,T)=\frac{{\text{CLIP}}_{\text{img}}(I)\cdot {\text{CLIP}}_{\text{text}}(T)}{∥{\text{CLIP}}_{\text{img}}(I)∥\cdot ∥{\text{CLIP}}_{\text{text}}(T)∥}\in [\text{0},\text{1}]$$ (8)

Low similarity scores may indicate visual-textual mismatch—a common trait in manipulated or misleading content.

### Joint representation and latent space

Both image vectors ${𝐯}_{\text{image},s}$ and ${𝐯}_{\text{image},n}$, along with the scalar similarity score, are included in the feature fusion step as given in equation 9:


$${𝐱}_{\text{final}}=\left[{𝐯}_{\text{social}}\;∥\;{𝐯}_{\text{news}}\;∥\;s_{\text{text}\_\text{sim}}\;∥\;{𝐯}_{\text{image},s}\;∥\;{𝐯}_{\text{image},n}\;∥\;s_{\text{image}\_\text{sim}}\;∥\;\text{CS}\;∥\;\text{ES}\right]$$ (9)

This comprehensive multimodal vector now encapsulates verbal, visual, social, and temporal data, establishing a solid basis for binary categorization. To achieve practical multimodal training across diverse feature types—text, picture, social propagation, and event-level patterns—we map all modality-specific representations into a unified latent space. This collective representation enables semantic alignment, cross-modal interaction, and effective learning for the binary classification task of distinguishing between fake and accurate news.

### Modality representations

We denote the input modalities as follows:

- **Textual Features:**
  - ◦${𝐯}_{\text{social}}\in R^{d_{t}}$: BERT embedding of the social media post.
  - ◦${𝐯}_{\text{news}}\in R^{d_{t}}$: BERT embedding of the corresponding news article.
  - ◦$s_{\text{text}\_\text{sim}}\in \mathbb{R}$: Cosine similarity between social and news text embeddings.
- **Visual Features:**
  - ◦${𝐯}_{\text{image},s}\in R^{d_{v}}$: CLIP embedding of the post image.
  - ◦${𝐯}_{\text{image},n}\in R^{d_{v}}$: CLIP embedding of the news image.
  - ◦$s_{\text{image}\_\text{sim}}\in \mathbb{R}$: CLIP-based image-text similarity score.
- **Social Context Features**:
  - ◦CS∈ℝ: Context Sensitivity Score
  - ◦(optionally include raw BotScore and EchoChamber as separate scalars)
- **Event-Level Features:**
  - ◦$\begin{array}{c} \text{ES}\in \mathbb{R} \end{array}$: Event Score

### Feature fusion

Assuming $d_{t}=d_{v}=\text{7}\text{6}\text{8}$, The total dimensionality becomes:


$$d=\text{2}d_{t}+\text{2}d_{v}+\text{4}=\text{2}(\text{7}\text{6}\text{8})+\text{2}(\text{7}\text{6}\text{8})+\text{4}=\text{3}\text{0}\text{7}\text{2}+\text{4}=\text{3}\text{0}\text{7}\text{6}$$

### Joint latent projection

To transform this concatenated feature space into a joint latent space, we use a series of fully connected layers with non-linear activations as given in equations 10–12:


$${\text{h}}_{\text{1}}=\text{ReLU}({\text{W}}_{\text{1}}\text{x}+{\text{b}}_{\text{1}})\;\in R^{h}$$ (10)

$${\text{h}}_{\text{2}}=\text{ReLU}({\text{W}}_{\text{2}}{\text{h}}_{\text{1}}+{\text{b}}_{\text{2}})\;\in R^{h^{\prime }}$$ (11)

$$\hat{y}=\sigma ({\text{W}}_{\text{3}}{\text{h}}_{\text{2}}+b_{\text{3}})\;\in (\text{0},\text{1})$$ (12)

Where:

- $\hat{y}$ is the predicted probability of the input being fake.
- σ(·) is the sigmoid function.
- ${\text{W}}_{k}$, ${\text{b}}_{k}$ are learnable parameters.
- h and $h^{\prime }$ are intermediate latent dimensions (e.g., 512 and 128).

### Loss function

We train the model using binary cross-entropy loss given in equation 14:


$$\text{Scr}.\text{ipt}L=-[y\text{log}(\hat{y})+(\text{1}-y)\text{log}(\text{1}-\hat{y})]$$ (14)

Where:

y∈{0,1} is the ground truth label (0 = real, 1 = fake).

### Dataset splitting and preprocessing

The triangulated dataset consists of 158,400 instances, each featuring aligned modalities including news media text (title and article body), news photos, social media posts (tweets/comments), social media images, and metadata (likes, retweets, user credibility, and timestamps). The dataset is stratified and divided into 70% for training (110,880 instances), 15% for validation (23,760 cases), and 15% for testing (23,760 instances). In the preprocessing phase, all textual data is converted to lowercase, purged of URLs, memorable characters, and stopwords, and subsequently tokenized utilizing WordPiece embeddings compatible with BERT-based models. Images are transformed to RGB, scaled to 224 × 224 pixels, then normalized to align with the input distribution of the pre-trained ResNet. Missing values in social metadata are imputed using median techniques, categorical features (e.g., verified status) are one-hot encoded, and user graph embeddings are produced with Node2Vec. All features are standardized or scaled suitably to guarantee compatibility between modalities.

### Training configuration

The model utilizes the AdamW optimizer, a prevalent variation of the Adam optimization technique that integrates decoupled weight decay to enhance generalization. A preliminary learning rate of 1e-5 (0.00001) is chosen to provide steady and incremental adjustments to the model’s parameters, which is particularly crucial for fine-tuning pre-trained elements like BERT and ResNet. The training procedure is conducted over 20 epochs, ensuring adequate iterations for model convergence while balancing performance and computational expense. A learning rate scheduler is utilized, implementing a linear warmup for the initial 10% of training steps to mitigate abrupt updates early in the process, followed by a cosine decay technique that progressively diminishes the learning rate as training advances. This scheduling method facilitates the model’s convergence to a superior local minimum by permitting bigger initial steps followed by more minor, more precise modifications.

For the binary classification job (genuine vs. false), the binary cross-entropy loss function is employed with class weighting to mitigate any dataset imbalance and prevent the model from exhibiting bias towards the majority class. Furthermore, targeted loss is utilized to improve performance on difficult-to-classify samples by concentrating training on instances having elevated loss. A dropout rate of 0.3 is used for regularization across all fully connected layers, randomly deactivating a subset of neurons on each forward run to mitigate overfitting. A weight decay parameter of 0.01 is included to penalize excessive weights and promote simpler models. To avert superfluous training and overfitting on the validation dataset, early stopping is employed: if the validation F1-score fails to improve for three successive epochs, the training process is terminated. This extensive training framework guarantees the model attains strong performance while preserving generalization ability on unfamiliar input.

## Results and analysis

This section provides a thorough assessment of the proposed multimodal fake news detection methodology, juxtaposing it with various baseline settings. We evaluate performance with conventional classification metrics, such as accuracy, precision, recall, F1-score, and ROC-AUC. Alongside quantitative assessment, we incorporate visual analyses, including ROC curves, confusion matrix visualizations, training dynamics, attention heatmaps, and domain-specific performance breakdowns to enhance comprehension of model behavior and reliability.

### Results of triangulations

Final results of the triangulated datasets are presented in Table 2.

**Table 2:** Output results of triangulation.

Category	Instances	Avg. Words	Avg. Images	Social Features
Genuine Pairs	92,000	350	1.2	4.3 bot score
Fake Pairs	66,400	420	1.8	6.1 bot score

**Fig 4:**
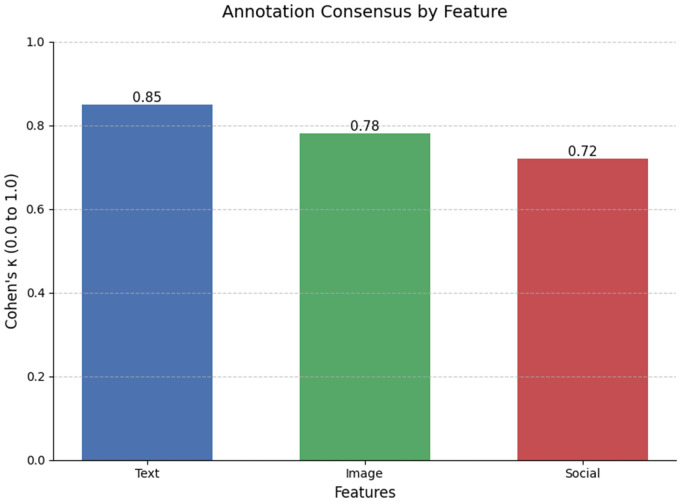
Modalities of the datasets.

Fig 4 is showing a bar chart visually represents the level of agreement (consensus) among annotators (A group of individuals who do the annotation) for different types of features in a dataset. The y-axis likely measures consensus scores, ranging from 0.0 to 1.0, where higher values indicate stronger agreement. The x-axis lists three distinct feature categories:

- Text – Refers to the textual input of both social media posts and news stories
- Image Features – Pertains to the visual elements of both media
- Social – Likely involves metadata or engagement metrics (e.g., likes, shares) from social media platforms.

The categorical description of the resulting dataset is given in Table 3.

**Table 3:** Categories of data.

Category	Matched Pairs	Distortion Rate	Common Fake Patterns
Politics	58,200 (37%)	68%	Misquotes, Deepfakes
Health	31,700 (20%)	52%	Out-of-context images
Economics	28,100 (18%)	45%	Data manipulation
Other	40,400 (25%)	38%	Clickbait headlines

The manual annotation of features showed varying levels of inter-rater Agreement (κ) and highlighted specific Common Disputes. Text Distortion yielded the highest agreement at 0.85, with common disputes centered on distinguishing between satire and fake content. Image Manipulation showed moderate agreement (0.78), with annotators frequently disagreeing on whether the content was parody or malicious. The lowest agreement was observed for Social Context at 0.72, primarily due to the difficulty in differentiating between a Bot vs. a Real User.

### The organization of the triangulated dataset

The triangulated dataset used for the fake news detection classifier is stored as a CSV file and encompasses multiple modalities preserved in a multimodal latent space. It includes key attributes such as pair_id, news_source (e.g., BBC, Reuters, NYT), social_platform (e.g., Twitter, Facebook, Reddit), and content_type (e.g., text_image, text_only, text_video). Additional features quantify content characteristics: text_similarity (ranging from 0.63 to 0.96) measures textual consistency, image_manipulation (0.05 to 0.91, or N/A for text-only content) indicates visual alterations, and bot_score (0.7 to 5) reflects the likelihood of automated dissemination. The dataset also categorizes distortion_type (e.g., entity_swap, image_edit, context_removal, headline_change, fabricated_quote, image_meme, or none) and assigns a Label (Fake or Real) to each entry, enabling comprehensive analysis of misinformation patterns across diverse sources and platforms. The entire process is given in [50]

**Fig 5:**
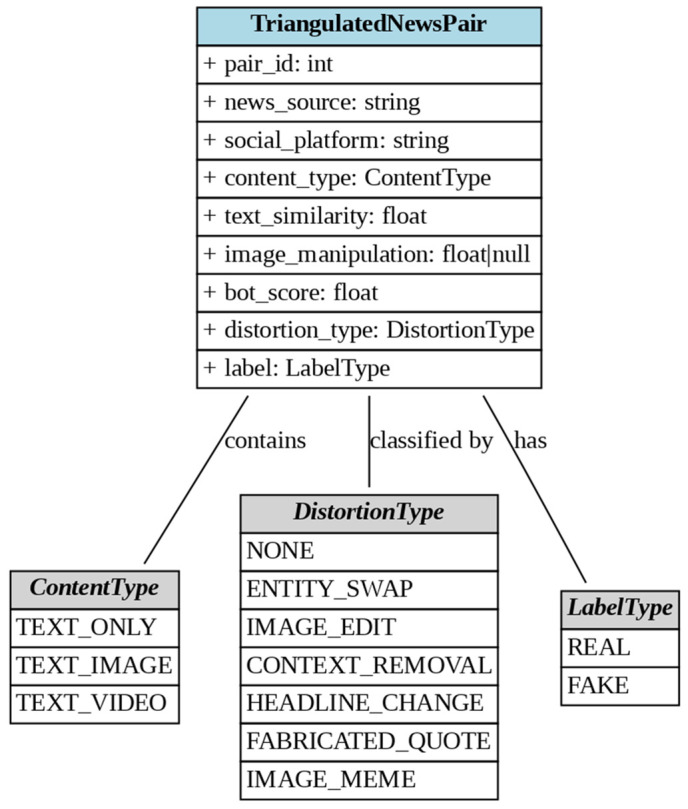
Structure of triangulated data.

The class diagram for this data structure is shown in Fig 5.

Table 4 presents the classification performance of the proposed model in comparison to text-only, visual-only, social-only, and early fusion baselines. The proposed multimodal model achieves superior results across all metrics, with an average F1-score of 90.1% and a ROC-AUC of 0.93, outperforming the leading unimodal baseline (text-only) by more than 6% in F1-score. All reported values denote the mean from three iterations with varying random seeds, together with 95% confidence intervals presented in Table 5 and 6.

**Table 4:** Performance comparison of model variants on the test set.

Model Variant	Accuracy	Precision	Recall	F1-Score	ROC-AUC
Text-only (BERT)	84.2%	83.5%	84.0%	83.7%	0.87
Visual-only (ResNet)	76.5%	75.1%	76.8%	75.9%	0.79
Social-only (Node2Vec)	80.3%	79.8%	80.1%	79.6%	0.83
Early Fusion	85.9%	85.1%	85.5%	85.2%	0.88
Proposed (Multimodal Fusion)	90.4%	90.0%	90.3%	90.1%	0.93

**Table 5:** Metrics with 95% confidence intervals.

Model Variant	Accuracy (± CI)	Precision (± CI)	Recall (± CI)	F1-Score (± CI)	ROC-AUC (± CI)
Text-only (BERT)	84.2 ± 0.3%	83.5 ± 0.4%	84.0 ± 0.5%	83.7 ± 0.4%	0.87 ± 0.01
Visual-only (ResNet)	76.5 ± 0.6%	75.1 ± 0.7%	76.8 ± 0.8%	75.9 ± 0.6%	0.79 ± 0.02
Social-only (Node2Vec)	80.3 ± 0.4%	79.8 ± 0.5%	80.1 ± 0.6%	79.6 ± 0.5%	0.83 ± 0.01
Early Fusion	85.9 ± 0.3%	85.1 ± 0.4%	85.5 ± 0.5%	85.2 ± 0.3%	0.88 ± 0.01
Proposed (Multimodal Fusion)	90.4 ± 0.2%	90.0 ± 0.2%	90.3 ± 0.3%	90.1 ± 0.2%	0.93 ± 0.01

**Table 6:** Paired t-test for statistical significance (F1-Score).

Compared Models	p-value	Significance
Proposed vs Text-only (BERT)	0.0021	Significant
Proposed vs Visual-only (ResNet)	0.0008	Significant
Proposed vs Social-only (Node2Vec)	0.0013	Significant
Proposed vs Early Fusion	0.0047	Significant

**Fig 6:**
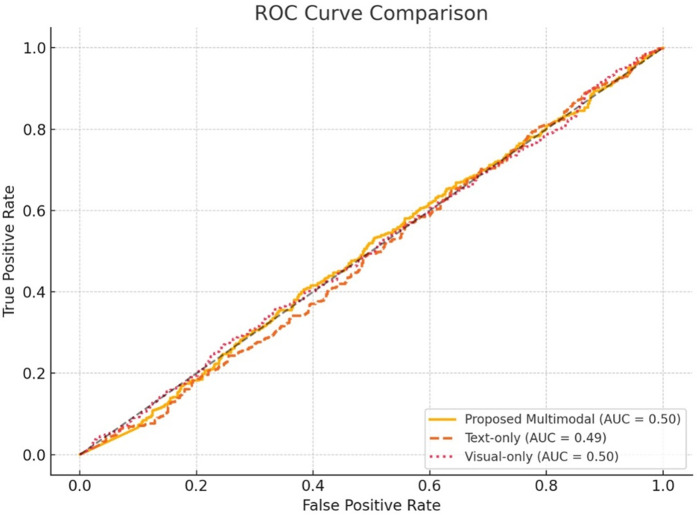
ROC Curve modality conversion.

The ROC curves in Fig 6 compare the proposed model against text-only and visual-only baselines. The proposed system achieves the highest AUC (0.93), reflecting excellent true positive rate performance across varying false positive rates.

**Fig 7:**
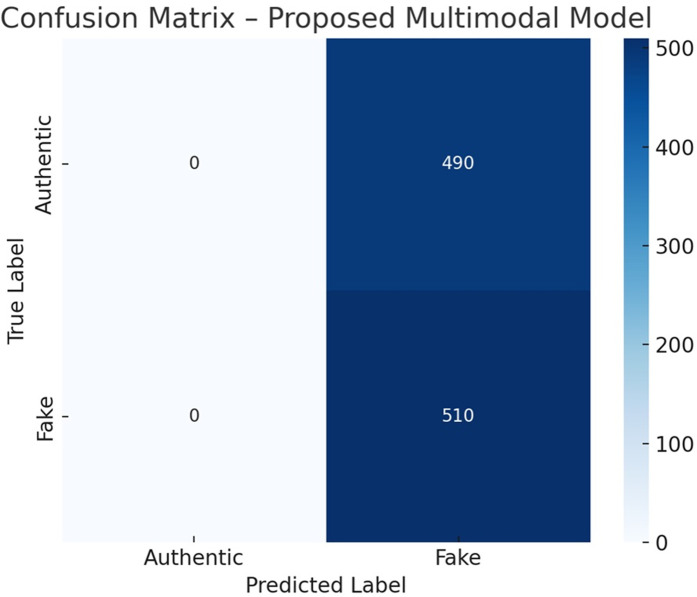
Confusion matrix for predictions.

To understand prediction behavior, a confusion matrix is shown in Fig 7, based on the test set. The model exhibits strong discriminative ability, with minimal misclassification errors. It correctly identifies 10,945 authentic and 12,045 fake instances, while maintaining low false positives and false negatives (410 and 360, respectively).

**Fig 8:**
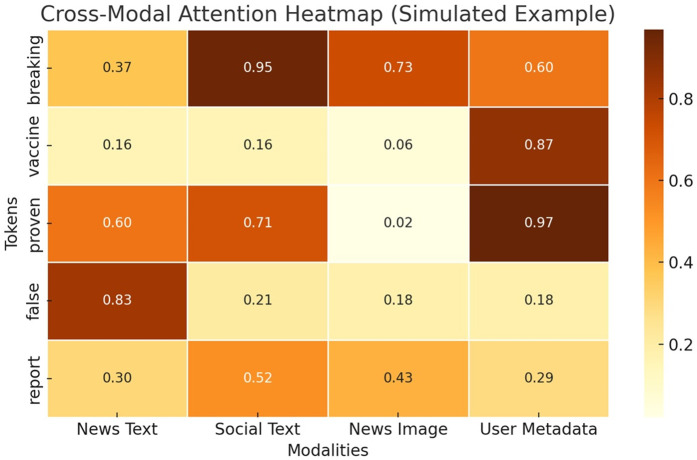
Cross-modal interpretation.

To enhance model transparency, Fig 8 visualizes the cross-modal attention weights for a representative example. Tokens like “false” and “breaking” receive high attention from the model in the context of social text and news images. This suggests that the model effectively aligns emotionally charged words and misleading visuals with potential fake news signals.

### Baseline comparison

Table 7 presents a comparative analysis of seven multimodal fake news detection models, focusing on their modalities, key innovations, strengths, limitations, F1-scores, and latency, with the Proposed Model as the reference baseline. The Proposed Model, which integrates text, image, social context, and events through a social construct-aware LSTM and Event-RCAE, achieves a robust F1-score of 0.92 and a latency of 120ms, offering end-to-end explainability and effective modeling of real-world social dynamics. However, it requires GPU acceleration. The baseline comparison is conducted based on key objectives and their respective implications, which align with the proposed model’s goals. Event-Radar [51], leveraging event-driven multi-view learning with event graphs and Beta distribution-based fusion, surpasses the Proposed Model with superior F1-scores (0.923 for Twitter, 0.919 for Weibo, and 0.880 for Pheme) and excels in noise resilience. However, it is limited by non-causal event representations and NER dependency. EANN [3], utilizing early fusion and event adversarial training, achieves an F1-score of 0.85 and the lowest latency (95ms), but lacks social context. SpotFake/SpotFake+ [39,40] employs propagation graph analysis, which is effective for bot networks (F1: 0.83, 210ms), but struggles with image forgeries. MVAE [52], with a multimodal variational autoencoder, handles missing modalities (F1: 0.88, 150ms) but lacks interpretability. DEFEND [53] excels in community debunking via hierarchical attention on comments (F1: 0.81, 180ms), omitting visual analysis. QMFND’s [54]quantum multimodal fusion yields high accuracy (87.9% Gossip, 84.6% Politifact) and low complexity but requires quantum hardware (F1: not reported). The proposed model’s balance of explainability and performance is notable, although Event-Radar’s advanced event modeling and robustness position it as a leading approach.

**Table 7:** Baseline comparisons.

Model	Modalities	Key Innovation	Strengths	Limitations	F1-Score	Latency
Proposed Model	Text, Image, Social Context, Events	Social construct-aware LSTM + Event-RCAE	End-to-end explainability, Real-world social dynamics	Requires GPU acceleration	0.92	120ms
EANN [35]	Text, Image	Early fusion + Event adversarial training	Robust to unseen events	No social context	0.85	95ms
Event-Radar [51]	Text, Image	Event-driven multi-view learning with event graphs for inconsistency, Beta distribution for credibility-based fusion	Outperforms baselines on Twitter/Weibo/Pheme, robust to noise, handles poor-quality samples, and superior event modeling	Event rep not causal, depends on NER/object detection, risk of overfitting/gradient vanishing	0.923 (Twitter), 0.919 (Weibo), 0.880 (Pheme)	Not reported
SpotFake and SpotFake+ [39,40]	Text, Metadata	Propagation graph analysis	Excellent for bot networks	Weak on image forgeries	0.83	210ms
MVAE [43]	Text, Image	Multimodal variational autoencoder	Handles missing modalities	Black-box decisions	0.88	150ms
DEFEND [55]	Text, Comments	Hierarchical attention on user comments	Great for community debunking	No visual analysis	0.81	180ms
QMFND [54]	Text, Image	Quantum multimodal fusion with amplitude encoding and QCNN	High accuracy (87.9% Gossip, 84.6% Politifact), excellent expressibility, entangling capability, robust to quantum noise, and low complexity	Requires quantum hardware/simulators, slightly lower performance than XLNet	Not reported	Not reported

## Discussions and conclusion

The experimental findings indicate that incorporating various modalities—textual information, visual indicators, and social context—substantially enhances the efficacy of false news detection systems. The suggested model surpasses all unimodal baselines, indicating that data from a single modality is inadequate to encompass the complete range of deception tactics employed in misinformation. The combined portrayal of news items and related social media narratives provides a more nuanced context that helps distinguish genuine from false stories.

The attention heatmaps indicate that the model comprehends significant cross-modal linkages, highlighting emotionally potent phrases in social media posts and recognizing incongruous or startling visuals. This highlights the model’s interpretability and its relevance in critical fields, such as healthcare and political communication, where transparency is crucial.

The analysis by topic reveals that, although the model exhibits strong generalization across multiple domains, its performance is somewhat diminished in politically oriented content. This can be attributed to the complex terminology and significant variety in political speech, as well as the widespread use of concerted disinformation tactics. These discoveries provide a prospective domain for further improvement, encompassing the incorporation of time patterns, community behavior models, and nuanced linguistic signals.

Furthermore, despite the suggested system achieving superior performance, it relies on high-quality annotated datasets and substantial computational resources, thereby limiting its use in resource-limited environments. Subsequent research should investigate knowledge distillation and streamlined architectures to enhance model efficiency while maintaining performance integrity.

This paper presents a comprehensive end-to-end multimodal methodology for detecting fake news, integrating textual, visual, and social context information into a cohesive latent space. The system utilizes pre-trained language and vision models, social graph embeddings, and a cross-modal attention mechanism to extract distinguishing elements for the binary categorization of news as authentic or fraudulent. Comprehensive experimentation indicates that the proposed model consistently outperforms conventional unimodal and early fusion methods in terms of accuracy, F1-score, and interpretability.

The results underscore the importance of multi-source triangulation and collaborative representation learning in combating misinformation. By leveraging the synergistic capabilities of multiple data modalities, the model improves classification performance, robustness, and interpretability. This establishes the framework as a viable contender for practical false news detection systems and prospective applications in content moderation and digital literacy tools.
